# Progress in Disease of Digestive Organs

**Published:** 1902-04-12

**Authors:** 


### Progress in Disease of Digestive Organs.

(*Continued from page* 11.)

**Syphilis of the Stomach.**—W. Soltan Fenwick[12] discusses the much neglected syphilitic diseases of the stomach. These include gummata, endarteritis, and chronic inflammation of the mucous membrane. A gumma may produce an irregular ulceration of the stomach and, in the infant, of the intestines also. Endarteritis leads to chronic ulceration or dyspepsia, followed by hæmatemesis. However, not more than 5 per cent. of ulcers of the stomach have any connection with syphilis. These are generally in males between 25 and 40 years of age, and are marked by anæmia, extreme severity of the pain, and vomiting, infrequency of hæmorrhage, resistance to the ordinary treatment of ulcer, and a tendency when cured by anti-syphilitic remedies to relapse. Great emaciation and pain which is worse at night, are also in favour of syphilis. The diagnosis from cancer is difficult; but a tumour and blood in the vomit are against syphilis, while the pain of cancer of the stomach is made worse by mercury and iodides. The gastric crises of tabes differ in occurring only at irregular intervals and are little affected by food. The treatment for syphilitic ulcers comprises absolute rest, diluted or peptonised milk, blisters on the epigastrium, and above all mercury combined with iodides. Lavage may be use for chronic gastritis, and it should not be forgotten that gastric irritation in a syphilitic patient may be due to the irritation of the medicine and not to specific ulceration.

**Gastric Physiology.**—Some curious discoveries have been made in the Lausanne laboratory[13] showing the mechanism by which the stomach secretes the quantity of acid or of pepsin required for each meal. Certain substances when ingested can cause an increase of the substance needed. Thus ethyl alcohol or a small quantity of Liebig's extract will greatly increase the secretion of H.Cl., while dextrin given per rectum or in small doses by the mouth increases the amount of pepsin. *Rumination* has been observed by S. Silvestri[14] in a father and three sons who had a neurotic family history. The habit commenced in the boys at about the age of puberty. The food was returned to the mouth in the order in which it had been swallowed, and was remasticated, the process lasting about two hours. By a powerful effort of will it was generally possible to stop the process. Silvestri regards it as a motor neurosis of the stomach, depending on a cortical affection and accompanied by a highly sensitive state of the gastric mucous membrane. Levi and Lolli[15] by accurate experiments show the great difference between digestion when an animal is fatigued and when it is fresh. The quantity of digestive juice is decreased in proportion to the exercise taken, the H.Cl. being specially lessened, and the time required to digest a quantity of albumen being greatly extended. Thus cubes of albumen, dissolved in 2½ to 3½ hours in a state of rest, required 8 to 11 hours with juice secreted after exercise. These tests were made by the method of Morner and Sjogvist, the older methods having been shown to be unreliable.

**Treatment.**—A form of dyspepsia associated with dryness of the nasal and pharyngeal mucous membrane, pallor, and absence of its secretion, together with a red, dry, and sometimes fissured tongue, is regarded by W. Stuart Low[16] as due to the deficiency of mucus and loss of its protective powers. He finds that great relief in these cases is obtained by giving tablets of dried mucin and bicarbonate of soda, 5 grains of each. Three of these may be given before and three more after each meal in severe cases and the amount gradually reduced. The nasal trouble may be treated by a wash made from the same materials. In the treatment of gastric ulcer Lauder Brunton[17] advises rest in bed with rectal feeding for a few days, next a tablespoonful of milk with a tablespoonful of limewater every two hours, the lime to be gradually replaced by milk, custard, and then pounded fish, pounded chicken and chocolate may follow, and afterwards patent foods. Suprarenal extract is recommended by W. Soltan Fenwick[18] for hæmatemesis. He gives 20 grains of the desiccated gland in 10 ounces of water as soon as possible after an attack, and repeats it after two hours. He finds this invaluable in recent ulcers and other affections, though hard chronic ulcers are probably but little helped by any hæmostatic. The extract is also very useful in gastric or rectal cancer.

**Duodenal Ulcer.**—Carter and Franklin[19] discuss three cases of perforated ulcers, in addition to 59 which are elsewhere recorded. They remark that of 32 operated upon 11 recovered. In one of these cases there had been a recent operation for perforated gastric ulcer, but a few days afterwards death from general peritonitis occurred, and a perforated duodenal ulcer was found. In another it followed an operation for appendicitis, and in a third it came on six months after an operation for stone in the kidney. The authors remark on the many cases which have followed operations in various parts of the body, and compare them with ulcers found after burns.

**Carcinoma of Duodenum.**—W. Soltan Fenwick[20] finds that this occurs in London only once out of 1,500 to 2,000 necropsies, that is, it is 20 times as rare as gastric cancer. If the growth is near the pylorus the diagnosis from gastric cancer is difficult; if it involves the orifice of the bile duct there may be liver symptoms, if below it there may be bilious vomiting and pancreatic juice in the vomit. The average duration of his cases was seven months. He warns us to distinguish cases from (*a*) pyloric stenosis due to simple ulcers; (*b*) from cancer of the pylorus, but here it is said there is more often absence of H.Cl., rarely bile in the vomit or diarrhœa alternating with constipation, and the tumour, if present, is nearer the middle line; from (*c*) tumours pressing on the duodenum; (*d*) cancer of the head of the pancreas; (*g*) gall-stones; and (*e*) from simple ulcer of the duodenum. The cachexia, wasting, vomiting, and dilatation of the stomach are among the most important general symptoms.

**Gall-stones** most frequently pass down the bowel and out by the rectum, but occasionally they lead to an abscess which opens on the skin or into the stomach. At times they pass into the stomach by a retroperistaltic wave, and may then be vomited. Kellett Smith and W. Crooke[21] record two instances in which stones of 5 and 13 grains respectively were vomited after attacks of biliary colic. The pylorus will sometimes allow the passage of much larger calculi than would seem possible.

**Epidemic Jaundice.**—An outbreak of about 150 cases occurred in Derbyshire, and one patient developed acute yellow atrophy, which was verified post-mortem. Another group of 150 cases appeared at Farnworth. These are probably the most extensive outbreaks which have happened in this country for some time.[22] The attacks varied from the mildest catarrhal jaundice to fatal ones presenting atrophy. The actual poison is unknown, but it appears to act on the smallest bile ducts, and not by causing catarrh of the common duct. As Dr. Peek points out, there is an incubation period of six or seven days, high fever and jaundice on the third or fourth day. The attack begins with a gastro-intestinal disturbance, and the infection is conveyed by individuals even to a distance, but cannot be traced to food or drink.

[12] Lancet, Sept. 28. [13] Lancet, Sept. 28. [14] Lancet, Nov. 16. [15] Brit. Med. Jour., Nov. 9. [16] Lancet. Oct. 12. [17] Brit. Med. Jour., March 1. [18] Brit. Med. Jour., Nov. 30. [19] Lancet, Nov. 2. [20] Edinb. Med. Jour., Oct. [21] Lancet, Dec. 7. [22] Brit. Med. Jour., Dec. 14.

